# Immunohistochemistry effect on diagnostic reliability for paediatric cancer at Mwanza region, Tanzania: a laboratory descriptive study

**DOI:** 10.3332/ecancer.2024.1809

**Published:** 2024-12-05

**Authors:** Jeffer O Bhuko, Erius Tebuka, Oscar Ottoman, Kristin Schroeder

**Affiliations:** 1 Pathology Department, School of Medicine, Catholic University of Health and Allied Sciences, Mwanza, Tanzania; 2 Oncology Department, Bugando Medical Centre, Mwanza, Tanzania; 3 Oncology Department, Duke Medical Centre, Durham, NC, USA

**Keywords:** biomarkers, diagnosis, immunohistochemistry, pathology

## Abstract

**Introduction:**

In nations with poor and intermediate incomes, cancer is one of the main causes of mortality. Immunohistochemistry (IHC) is crucial for an accurate cancer evaluation, prognosis and treatment decision-making. To use IHC, a significant amount of facilities and capacity growth are needed. Because of this, it is crucial to comprehend the potential effects of IHC and identify the most essential reagents required to distinguish between typical diagnoses in our environment.

**Objective:**

Employing IHC, this study aims to assess how well paediatric cancer diagnoses in Tanzania can be made and to identify the most widely used biomarkers for diagnostic distinction.

**Methods:**

Pathology samples from kids who were given cancer diagnoses in 2018 at the Bugando Medical Centre in Mwanza, Tanzania, were examined using H&E staining. Basic demographic information from the histology form was gathered in addition to the reported histopathology results from Bugando Medical Centre, including patient age, sex and sample collection. Muhimbili National Hospital received tissue from the histology block for the IHC examination. It was determined which reagents/biomarkers were required for diagnostic confirmation by comparing the histopathology data for diagnostic agreement, variations in diagnosis and other factors.

**Results:**

The examinations included 105 (105) patients with paediatric cancer. 55.2% of the population, who had a median age of 6 years (IQR 3–9), were female. Burkitt and NHL-DLBCL were the paediatric diagnoses with the greatest pathology. The correlation between H&E and IHC histology was 51.0%. 17.6% (*n* = 18) of diagnoses had enhanced diagnostic specificity (e.g., NHL to diffuse large B cell lymphoma), and 31.4% of diagnoses were altered as a result of IHC.

**Conclusion:**

Considering that the diagnosis of juvenile cancer changes in about 30% of cases, IHC is crucial for accurate diagnosis. IHC retraining is crucial, and developing nations can successfully adopt a modest shared biomarker panel to improve therapy allocation.

## Background

Among 250,000 new cases of cancer diagnosed every year, only about 20%–30% of paediatric cancers are diagnosed [1]. A significant gap in diagnosis and treatment exists between low middle-income countries (LMICs) and high-income countries (HICs), despite the importance of paediatric oncology care which has shown an increase in survival in HIC [2]. In the USA 80% of paediatric cancer patients survive [3], while in LMIC, especially from Sub-Saharan African countries less than 25% of paediatric cancer patients survive [4]. Many setbacks exist in effective paediatric cancer treatment in LMIC including late-stage presentation, limited radiotherapy and common chemotherapy and cost of treatment [5]. Furthermore, despite the presence of all these setbacks, problems in inefficient healthcare and palliative care result in a poor prognosis for paediatric cancer in LMIC. The significance of cancer therapies that target the specific biomarkers for paediatric oncogenes, such as CD31, Desmin and Myogenin for Ewing’s Rhabdomyosarcoma, CD15 and CD 30 for Hodgkin lymphoma, CD3 for B cell lymphoma, CD20 for T cell lymphoma, CD10 for Burkitt lymphoma, all these biomarkers for confirmatory diagnostic purposes at any body part for a respective paediatric malignancy site. Immunohistochemistry (IHC) can be used for a better diagnosis and understanding of the origin of various tumour cells using various markers of immune response like human leukocyte antigen-DR and those expressed on monocytes and macrophages like CD 68 and leukocyte common antigen [6]. Cancer treatment relies much on the effectiveness of advanced diagnosis such as IHC and immunofluorescence techniques. Around 60 new paediatric patients are diagnosed with various cancers each year by cellular morphology pathology (H&E stain) at Bugando Medical Centre (BMC) hospital in the United Republic of Tanzania. The total number of new paediatric diagnosed with cancer annually is approximately 210 paediatric patients in Tanzania countrywide [7]. Patients receive diagnosis and treatment from BMC, and a few of them are transferred to Muhimbili National Hospital (MNH) for further diagnosis and treatment.

The current study evaluates the significant effects of IHC on diagnostic accuracy for paediatric cancer in Tanzania compared to the routine H&E staining cancer identification. The most often utilised IHC markers for differentiating paediatric cancer patients have been identified, and IHC diagnosis has been able to find numerous biomarkers that are in charge of capturing particular cancer antigens for the accurate treatment of cancer. Abundance biomarkers of cancers should be known around the regions and the impact magnitude of IHC diagnosis results among paediatric cancer patients is not known. The study brought to attention the necessary biomarkers to be used for paediatric cancer patients, the rule in the wanted biomarkers for cost-effectiveness purposes and the impact magnitude the IHC diagnostics results will add on routine tissue Pathology (microscopy) at BMC. The current study evaluates the impact of IHC on diagnostic accuracy for paediatric cancer in Tanzania and identifies the most common biomarkers used for diagnostic differentiation, and IHC diagnosis mainly for important paediatric biomarkers purchases to reduce IHC reagents cost for the whole batch set of paediatric.

## Methods

IHC or IHC is a method used in the laboratory to identify particular proteins or antigens in tissue sections. It is commonly utilised in pathology research and diagnostics purposes. The following are the guidelines for doing an IHC test in a laboratory: Safety adhered to the recommended personal protective equipment, which was gloves, laboratory coats and safety goggles. Sample Preparation: Using a microtome, tissue samples should be fixed, embedded in paraffin and cut into thin slices that were typically 4–5 mm thick. Make sure the tissue slices were adhered to the glass slides appropriately. Choose and validate antibodies that were specific to the desired protein. While secondary antibodies that have been marked with a visible marker (such as an enzyme or fluorescent dye) bind to the main antibody, primary antibodies bind to the target protein. Controls: Every IHC test should include both positive and negative controls. Negative controls lack the target protein, whereas positive controls were tissues that have been shown to express the target protein. Blocking: To lessen non-specific binding, saturate non-specific binding sites on the tissue by treating sections with a blocking substance (such as serum), antigen Retrieval: To reveal epitopes in some formalin-fixed tissues, antigen retrieval may be necessary. Enzymatic digestion or heat-induced epitope retrieval can be used to accomplish this. Staining Optimisation: To get the best signal-to-noise ratio, optimise the staining conditions (antibody concentration, incubation time and temperature).

H&E conventional staining principles: When working with paraffin-embedded tissues, deparaffinize the slides with xylene or another clarifying agent before the section is rehydrated with a graded series of ethanol. Hematoxylin is a simple dye that binds to nuclear material. Stain the tissue slices with it. To obtain a nuclear stain, make sure enough time was allowed for the staining process, usually a few minutes. Washed extra hematoxylin, rinsed the slides in distilled water, to attain the appropriate nuclear-stained intensity eliminate extra hematoxylin and differentiate the stained sections in an acid-alcohol solution. This process needed to be closely observed under a microscope. Re-rinsed the slides in water to stop the differentiating process after washing them, eosin is an acidic dye that stains extracellular matrix and cytoplasmic structures, thus counterstaining the sections with it. Dehydration was performed to eliminate extra water and get the slides ready for mounting, dehydrate them using a graduated alcohol solution. Clearing and mounting were performed to make the slides transparent, using a clearing agent like xylene to clear and prevent drying, using a mounting medium and coverslip. Analysis and Microscopy of a light microscope to examine the stained tissue sections, objectives with different magnifications (10xs, 40x and 100x). Examined tissue sample morphology and any pathogenic element that may be present.

The current study is a hospital-based descriptive laboratory-sectional study from January 2018 to July 2019. The study was conducted at BMC for tissue Pathology results and MNH for IHC diagnostic results. The study included paediatric cancer patients less than 18 years of age, who had both H&E and IHC performed on diagnostic tissue. Pathology samples for children diagnosed with cancer at BMC (Mwanza, Tanzania) in 2018 were evaluated using H&E staining. The sample size paediatric biopsies of 105 [8] from Schroeder *et al* [8] in Northern Tanzania where BMC is located. Calculated from the Kish Leslie formula. H & E staining from BMC was used for IHC staining at MNH from the simple random sampling procedures. Basic demographic information from the histology form was recorded, including patient age, sex and sample collection, as well as the reported H&E results. The tissue was sent to MNH in collaboration with the pathologist team from Crumlin Hospital Ireland for IHC review. The histopathology results were compared for diagnostic agreement, change in diagnosis and identified which biomarkers were necessary for diagnostic confirmation.

A structured review was done on the agreement diagnosis, additional diagnosis, new diagnosis and reagents/biomarkers identified for specific paediatric cancer patients.

## Results

### Social demographic

One hundred and five paediatric cancer patients were reviewed. 58 (55.2%) were female with a median age of 6 years (IQR 3–9 years). The most common paediatric diagnoses were Burkitt lymphoma and Non-Hodgkin Lymphoma specifically Diffuse Large B-cell lymphoma, anaplastic large cell lymphoma, follicular lymphoma (FL) and lymphoblastic lymphoma (LL).

### Concordant and discordant of diagnosis

Overall histology concordance between H&E and IHC was 51.0%. The most common discordant diagnosis was the NHL of ALCL (ALK1-negative, CD-45, 30-positivity). The most concordant was leukaemia (BCR-ABL).

Diagnostic specificity (example: in non-hodgkin lymphoma) improved by 17.6% (*n* = 18).

The most common diagnosis with increased specificity was DLBCL. Among NHL, 39% (*n* = 7) were ALCL, 27% (*n* = 5) DLBCL, 17% (*n* = 3) LL and 17% (*n* = 3) FL.

### Alteration of diagnosis

The use of IHC resulted in a change in diagnosis for 31.4% (*n* = 32) of patients. The most common diagnoses that were changed because of IHC were Burkitt lymphoma, Sarcoma and Necrosis, respectively.

## Discussion

IHC is critically important for the accurate diagnosis of paediatric cancer, with over 30% of all cases identified as having treatment-changing diagnoses among our cohort. The changes in diagnosis improved the outcome for the treatment of choice at BMC, the management regime was completely altered from the confirmatory diagnosis of IHC from MNH. A case example from confirmed acute leukaemia cases from flow cytometry results from MNH resulted in a complete remission and prevented the relapse of the disease to the paediatric confirmed diagnosis of specific acute leukaemia, the confirmed lymphoma cases of Hodgkin and non-Hodgkin lymphoma, and inflammatory reactions considered malignancy at BMC are managed accordingly. IHC training would be important to include in capacity development for paediatric cancer programmes in low-resource settings. Most common discordant NHL (especially ALCL, DLBCL), the main reason can be the presence of many sub-types of NHL; hence, specificity on the exact type of NHL without biomarkers becomes difficult meaning difficulties in distinguishing types of NHL on convectional histology (H&E staining).

A higher discordance resulting from the complete changes in diagnosis between the two diagnostic techniques from the BMC and MNH facilities is mainly attributed to missing IHC diagnostic reagents that could provide a significant range for specific and accurate diagnosis. Advanced tools for flow cytometry provide the best room for confirmation of leukaemia cases for accurate diagnosis scores and minimal residual disease. These are the reasons for the discordance in diagnosis between the two techniques and facilities. A study done by Jaffe *et al* [9] shows difficulties of NHL classification on histological appearance (H&E staining) alone not to be a reliable indicator and encouraged the use of advanced diagnosis markers (IHC, immunophenotyping).

A study done by Tran *et al* [10] shows there is high specificity in the diagnosis of Hirschprung’s disease using marker calretinin IHC compared to (H&E staining) on the rectal suction biopsies on frozen embedded tissue. Pileri *et al* [11] showed the identification and classification of neoplasm using different IHC markers CD68, Lysozyme, CD21, CD35 and S100 protein in the classification of these neoplasm high sensitivity and specificity rate was observed.

The importance of accurate paediatric cancer diagnosis is crucial at the beginning of the treatment to avoid further complications on the paediatric cancer advancement, poor prognosis and finally death due to mismanagement attributed to inaccurate diagnosis.

A study done by Proctor *et al* [12], an important expert shows discordance rate variation between 3.6% and 34.1%; the study demonstrated the importance of review in accurate diagnosis and timely lymphoid management.

We found that a limited common biomarker panel can successfully be used to confirm diagnosis among the paediatric cancer population. WHO recommended, on the common panel that 78 different paediatric cancer genes be identified by biomarkers, all 15 biomarkers present in this study were identified from the WHO paediatric cancer identification panel.

A study done by Hayes *et al* [13] from a report on AHOPCA Pathology, still reveals the struggle made in the anatomical pathology of LMICs. The training was offered a 5-day pathology training work to Pathologist and Histo-technologists from various LMICs of the Caribbean region. Assessment was made in the review and evaluation of the quality of IHC slides produced after the training course. A comparison was made between the training slides and the original sides from the institute, 5 days of training slides appeared effective. However, it is part of quality improvement and validation in the development of IHC in LMICs. Ottmann *et al* [14] on the detection of a mitotic figure in thin melanoma. IHC does not replace Hematoxylin and Eosin stain. A study found that comparing several mitotic figures has a slight variation which is not significant. IHC for mitotic figures could not replace H&E’s careful evaluation of slides. IHC detection is only an additional tool. A study was done by Nielsen *et al* [15] on Proliferation indices of phosphohistone H3 and Ki67 strong prognostic markers I/II melanoma. The finding from the study was phosphohistone H3/MART and Ki67/MART were strong and effective to Hematoxylin and Eosin stain, this presentation is similar to the study done at BMC on the impacts of IHC compared to H&E on paediatric cancer patients, these two IHC stains seem to be a robust alternative to conventional mitotic detection by H&E stain in melanoma. Study done by Mezheyeuski *et al* [16] the results were compared with those of conventional IHC, and related to corresponding RNA-sequencing expression values. We found a strong correlation between the visual and digital quantification of lymphocytes for CD45RO (correlation coefficient: *r* = 0.52), FOXP3 (*r* = 0.87), CD4 (*r* = 0.79), CD20 (*r* = 0.81) and CD8 (*r* = 0.90) cells. The conclusion was, that the fluorescence multiplexed IHC method, based on only one tissue section, provided reliable quantification and localisation of immune cells in cancer tissue.

The application of this technique to clinical biopsies can provide a basic characterisation of immune infiltrates to guide clinical decisions. The 30% (30 children out of 100) discordance diagnosis difference between the two techniques (H&E and IHC) is a discouraging result because having lower efficacy on a true finding could bring a proper and specific treatment regime. The discordant is not acceptable by the World Health Organisation in LMICs where diagnostics tools are limited, places a significant focus on improving access to reliable diagnostic methods and training health care professionals through capacity building.

## Conclusion

IHC is critically important to improve diagnostic accuracy for paediatric cancer in lower middle-income Countries based on the 31.4% of the changed diagnosis between IHC and convectional histology (H & E) staining among paediatric cancer patients.

## Conflicts of interest

The authors declare no conflicts of interest.

## Funding

The authors received no financial support for the research, authorship and/or publication of this article.

## Ethical approval

Ethical clearance certification number No. 016/2023. from the joint Catholic University of Health and Allied Sciences-Bugando Medical Centre: Research and Ethics Committee (REC).

## Author contributions

JB contributed to data collection for BMC pathology results and Muhimbili IHC results, writing of manuscript JB and KS. OO contributed to the design and implementation of the research, JB, diagnosis for BMC pathology results, OO, JB, diagnosis for Muhimbili IHC results, ET, design and implementation of the research, ET, diagnosis for Muhimbili IHC results, design and implementation of the research, KS, the analysis of the results and to the writing of the manuscript.

## Figures and Tables

**Figure 1.**
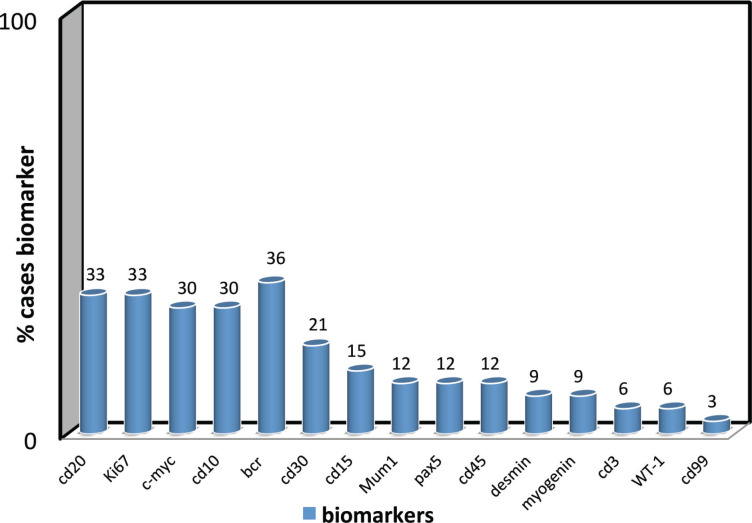
Biomarker confirmation frequency of biomarker use for diagnostic confirmation (cd20, Ki67, c-myc, cd10, bcr, cd30, cd15, Mum1, pax5, cd45, desmin, myogenin, cd3, WT-1, cd99).

**Figure 2.**
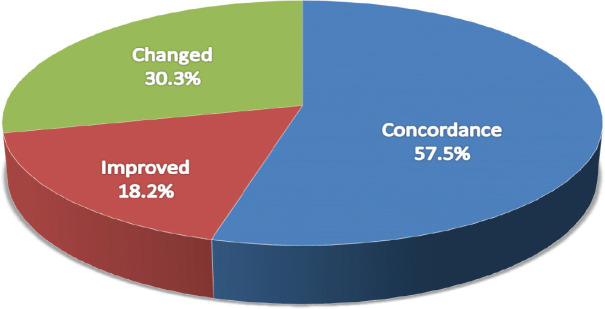
Impact of the diagnosis changes between H&E and IHC concordance between conventional pathology and IHC.

**Figure 3.**
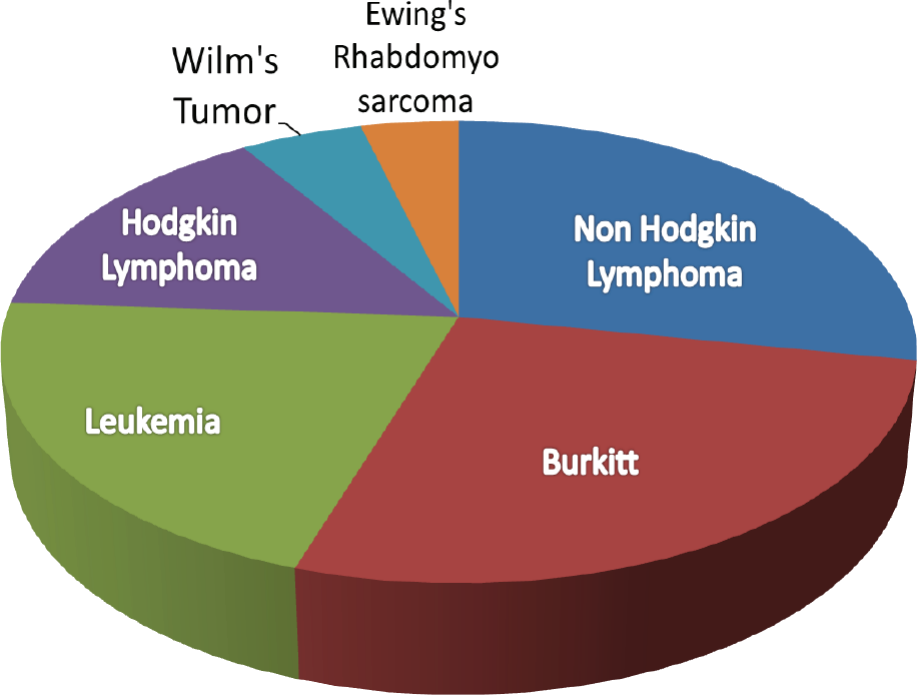
Paediatric cancers diagnosed at BMC annually the most common paediatric cancer convectional pathology diagnosis at BMC (*N* = 105).

**Table 1.** Annually cancer diagnosis between H& E stain and IHC comparison of paediatric cancers impacts for diagnosis at BMC annually (*N* = 105).

Comparison (H&E versus IHC)	Cancer/tumour diagnosed	Frequency (*n*(%))
Total concordant Dx, 55 (51%)	Leukaemia	16 (29%)
	BL	12 (22%)
	HL	10 (18%)
	Carcinoma	7 (12%)
	Sarcoma	6 (11%)
	Fibrosis	2 (04%)
	Inflammation	2 (04%)
Total specific (additional Dx required), 18 (17.6%)	ALCL	7 (39%)
	DLBCL	5 (27%)
	LL	3 (17%)
	FL	3 (17%)
Total changed Dx, 32 (31.4%)	BL	8 (25%)
	Sarcoma	6 (19%)
	Necrosis	6 (19%)
	Leukaemia	5 (16%)
	Inflammation	4 (12%)
	Carcinoma	2 (06%)
	Fibrosis	1 (03%)
